# Utilizing Nontraditional Data Sources for Near Real-Time Estimation of Transmission Dynamics During the 2015-2016 Colombian Zika Virus Disease Outbreak

**DOI:** 10.2196/publichealth.5814

**Published:** 2016-06-01

**Authors:** Maimuna S Majumder, Mauricio Santillana, Sumiko R Mekaru, Denise P McGinnis, Kamran Khan, John S Brownstein

**Affiliations:** ^1^ Computational Epidemiology Group Division of Emergency Medicine Boston Children’s Hospital Boston, MA United States; ^2^ Engineering Systems Division Massachusetts Institute of Technology Cambridge, MA United States; ^3^ School of Engineering and Applied Sciences Harvard University Cambridge, MA United States; ^4^ Department of Pediatrics Harvard Medical School Boston, MA United States; ^5^ Epidemico, Inc. Boston, MA United States; ^6^ Department of Medicine University of Toronto Toronto, ON Canada; ^7^ Li Ka Shing Knowledge Institute St. Michael's Hospital Toronto, ON Canada

**Keywords:** Zika virus disease, digital disease surveillance, mathematical modeling, reproductive number, transmission dynamics

## Abstract

**Background:**

Approximately 40 countries in Central and South America have experienced local vector-born transmission of Zika virus, resulting in nearly 300,000 total reported cases of Zika virus disease to date. Of the cases that have sought care thus far in the region, more than 70,000 have been reported out of Colombia.

**Objective:**

In this paper, we use nontraditional digital disease surveillance data via HealthMap and Google Trends to develop near real-time estimates for the basic (*R*_0_) and observed (*R*_obs_) reproductive numbers associated with Zika virus disease in Colombia. We then validate our results against traditional health care-based disease surveillance data.

**Methods:**

Cumulative reported case counts of Zika virus disease in Colombia were acquired via the HealthMap digital disease surveillance system. Linear smoothing was conducted to adjust the shape of the HealthMap cumulative case curve using Google search data. Traditional surveillance data on Zika virus disease were obtained from weekly Instituto Nacional de Salud (INS) epidemiological bulletin publications. The Incidence Decay and Exponential Adjustment (IDEA) model was used to estimate *R*_0_ and *R*_obs_ for both data sources.

**Results:**

Using the digital (smoothed HealthMap) data, we estimated a mean *R*_0_ of 2.56 (range 1.42-3.83) and a mean *R*_obs_ of 1.80 (range 1.42-2.30). The traditional (INS) data yielded a mean *R*_0_ of 4.82 (range 2.34-8.32) and a mean *R*_obs_ of 2.34 (range 1.60-3.31).

**Conclusions:**

Although modeling using the traditional (INS) data yielded higher *R*_0_ estimates than the digital (smoothed HealthMap) data, modeled ranges for *R*_obs_ were comparable across both data sources. As a result, the narrow range of possible case projections generated by the traditional (INS) data was largely encompassed by the wider range produced by the digital (smoothed HealthMap) data. Thus, in the absence of traditional surveillance data, digital surveillance data can yield similar estimates for key transmission parameters and should be utilized in other Zika virus-affected countries to assess outbreak dynamics in near real time.

## Introduction

Recent infectious disease outbreaks—including severe acute respiratory syndrome (SARS), Middle East respiratory syndrome (MERS), Ebola, and influenza A (H1N1)—have presented great challenges to the global public health community, including lack of basic epidemiologic knowledge to support important preparedness and control decisions. To address this gap, innovative surveillance methods have been developed over the last several years to leverage the increasing availability of digital data related to outbreaks. To date, many studies have retrospectively examined nontraditional digital data sources and have demonstrated their utility in estimating epidemic curves or changes in important epidemiologic parameters over time [1-3]. Such studies have provided a foundation for building near real-time prospective analytic techniques that can assess transmission dynamics in the absence of traditional data. These methodological developments fill a knowledge vacuum that may prove useful for public health decision making in the early stages of an outbreak.

The ongoing outbreak of Zika virus disease in Central and South America has attracted global attention due to its rapid geospatial growth as well as concerns over associated central nervous system complications [4,5]. Although Zika virus is primarily transmitted via *Aedes* mosquitoes, evidence of vertical and sexual transmission exists [6-8]. Likely introduced to the Americas in mid- to late 2013, the virus has since been propagated by the density of competent vectors throughout the region [8]. At present, approximately 40 countries in Central and South America have experienced local vector-borne transmission, resulting in nearly 300,000 total reported cases to date [9]. Of the cases that have sought care thus far in the region, about 70,000 have been reported out of Colombia, of which 17% were pregnant at time of clinical or laboratory diagnosis [9,10]. However, given the generally mild nature of Zika virus disease and subsequent lack of care seeking, reported cases undoubtedly comprise a small fraction of total cases [11,12].

Current prevention efforts focus on vector suppression [13], while interest in and efforts toward vaccine development are mounting rapidly due to increasing rates of Guillain-Barré syndrome following Zika virus infection and microcephaly in newborn babies born to women infected with Zika virus while pregnant [4,5]. Quantitative analyses designed to inform vaccine policies—in addition to other preparedness and control activities—are dependent on the transmission dynamics associated with the disease and, therefore, estimates for critical epidemiologic parameters are urgently needed for such decision making within the context of Zika virus disease.

In this paper, we use nontraditional digital disease surveillance data via HealthMap and Google Trends to develop near real-time estimates for the basic and observed reproductive numbers associated with Zika virus disease in Colombia as well as expected final outbreak size and duration. We then validate our results against traditional health care-based disease surveillance data and discuss the implications of our work on outbreak mitigation strategies in Colombia and assessment of transmission dynamics elsewhere in the region.

## Methods

**Figure 1:**
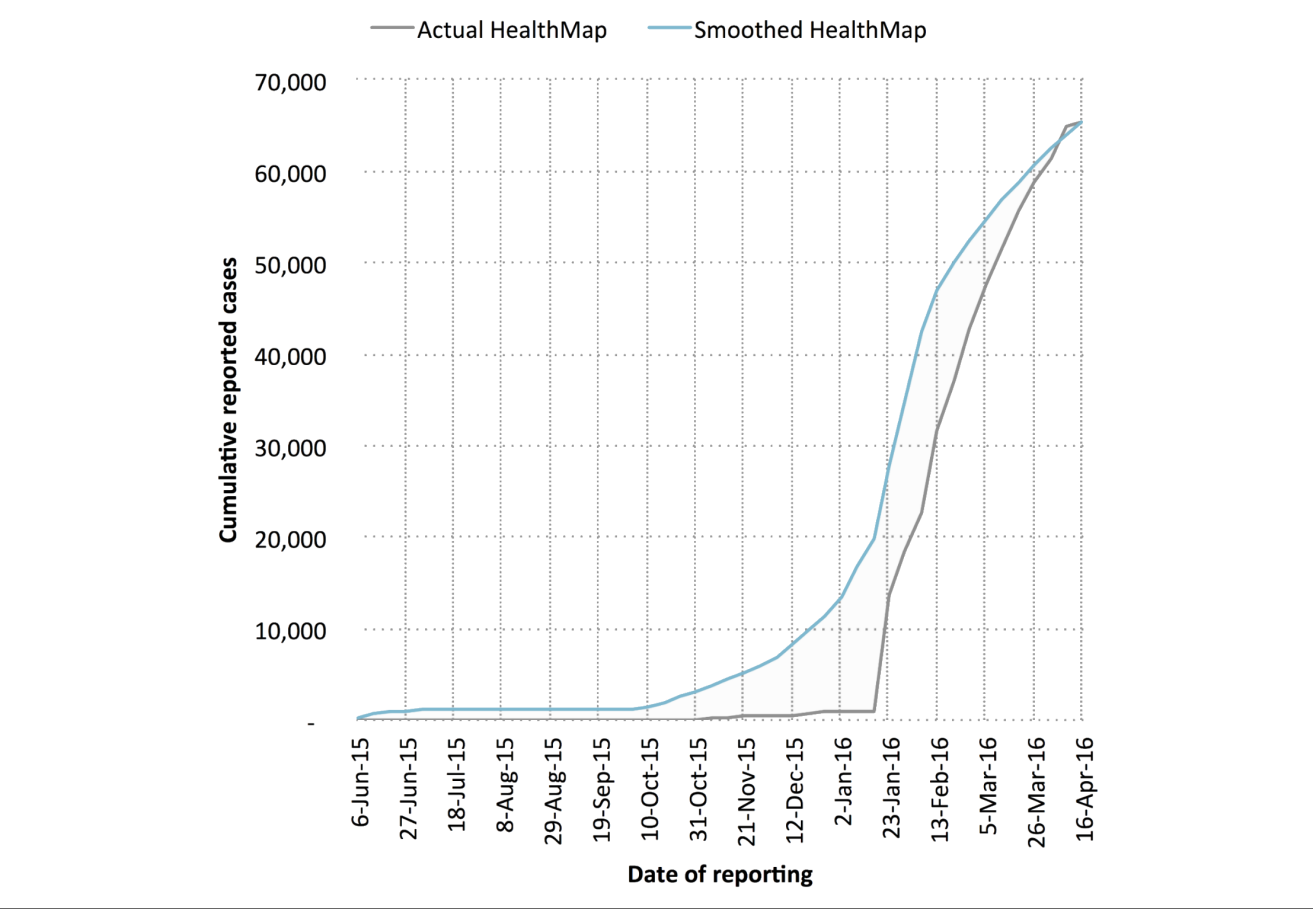
Cumulative case curve of Zika virus disease in Colombia as captured by the HealthMap digital disease surveillance system. Linear smoothing was conducted to adjust the shape of HealthMap cumulative case curve using Google search data.

Cumulative reported case counts of Zika virus disease in Colombia were acquired via the HealthMap digital disease surveillance system, consisting of 28 unique nongovernmental media alerts between October 16, 2015 and April 16, 2016 [14]. The cumulative reported case curve obtained from these reports shows an unrealistic L-shape, presumably due to increased interest in reporting during recent weeks and lack of awareness during early weeks (Figure 1). By assuming that the total number of cases obtained from HealthMap was a reasonable approximation of reality for the given time period, we used Google search data to distribute cumulative reported case counts more realistically over time.

Although many cases of dengue and influenza go undetected, previous studies have shown that relevant Google search trends demonstrate high linear correlation with reported disease incidence over time [15,16]. Thus, we obtained weekly Google search fractions of the term “Zika” from Colombia via the Google Trends website (accessed on April 29, 2016). These search fractions are displayed weekly as normalized values that range from zero to 100, which reflect the level of nationwide search interest in the word “Zika” from January 4, 2004 (first available datum) to April 16, 2016.

We created a smoothed cumulative incidence curve (referred to as “smoothed HealthMap”) by scaling the Google search curve against the HealthMap-reported Zika cases [17]. The scaling constant was obtained by dividing the most recent total number of HealthMap-reported Zika cases by the total number of Google search fractions from May 31, 2015 to April 16, 2016. Perhaps due to initial delays in reporting, the first relevant uptick of the term “Zika” in the Google Trends data occurred during the week of May 31, 2015, approximately 20 weeks before the first HealthMap alert of Zika virus disease in Colombia. Because of this, May 31, 2015 was selected as the start date for modeling efforts using smoothed HealthMap data; April 16, 2016 (last available datum at time of manuscript preparation) was selected as the cut-off date.

**Figure 2:**
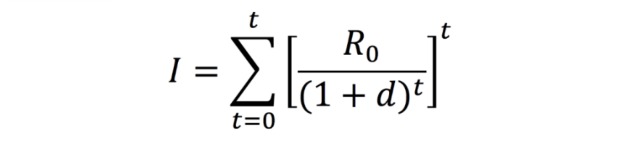
Cumulative incidence (*I*) expressed in terms of *R*_0_ and *d*.

Due to successful applications in other data-scarce (ie, cumulative incidence only) settings, the Incidence Decay and Exponential Adjustment (IDEA) model was used to estimate the basic reproductive number (*R*_0_) and the discount factor (*d*) associated with the ongoing outbreak [2,18,19]. Both *R*_0_ and *d* were solved for using nonlinear optimization to minimize the sum of squared differences (SSD) between reported (user-inputted) and modeled cumulative incidence (*I*) curves across multiple serial intervals (ie, outbreak generations). Figure 2 presents a formulation for *I* expressed in terms of *R*_0_ and *d*, where *t* is the number of outbreak generations (ie, serial intervals) that have passed thus far and is inversely proportional to the serial interval length (ie, number of days per serial interval [SI]). Given that distribution for the SI associated with Zika virus disease had not yet been established, *R*_0_ and *d* were solved for iteratively over a range of 14 deterministic lengths (10-23 days) [20].

These values of *R*_0_ and *d* were then used to define maximum, minimum, and mean values for the observed reproductive number (*R*_obs_), final reported outbreak size (*I*_max_), and final reported duration (*t*_max_).The observed number of secondary infections per infected individual for a given value of *t* (*R*_obs_) was calculated using the following equation: *R*_obs_ = *R*_0_/(1+ *d*)^*t*^.

When *d* is greater than zero, *R*_0_ does not equal *R*_obs_. In such circumstances, disease incidence is nonexponential due to either planned or unplanned reductions in disease duration, contact rate, or infectiousness of cases [18]. Likewise, final reported outbreak duration (*t*_max_) was calculated as follows [18]: *t*_max_≥ln(*R*_0_)/ln(1+ *d*).

**Figure 3:**
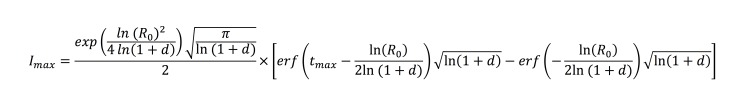
Final reported outbreak size (*I*_max_) expressed in terms of *R*_0_ and *d*.

Final reported outbreak duration can also be expressed in days by multiplying *t*_max_ by SI; however, in calculating *I*_max_, original units (ie, outbreak generations) are used (Figure 3).

In the event that a viable vaccine is developed before the ongoing outbreak in Colombia ends (*t*_max_), the following equation was used to assess the percentage of the susceptible population that would need to be immunized against Zika virus (%Vax) to eliminate transmission, assuming 100% vaccine efficacy: %Vax=1–(1/ *R*_obs_).

After completion of the analyses on the digital surveillance data, we performed a validation study using traditional surveillance data obtained from weekly Instituto Nacional de Salud (INS) (National Institute of Health, Colombia) epidemiological bulletin publications [21]. The INS first reported incidence of Zika virus disease in Colombia on October 16, 2015. However, subsequent publications indicated that the outbreak likely began during epidemiologic week 32 of 2015 or earlier [22]. As result, August 22, 2015 was selected as a start date for modeling efforts using INS data. April 16, 2016 (date of the most recent publication at time of manuscript preparation) was selected as the cut-off date [22]. The analyses described previously for the smoothed HealthMap dataset were conducted on the INS dataset as well, resulting in *R*_0_, *d*, *R*_obs_, *I*_max_, *t*_max_, and %Vax estimates for both digital (smoothed HealthMap) and traditional (INS) cumulative reported case data.

## Results

**Figure 4:**
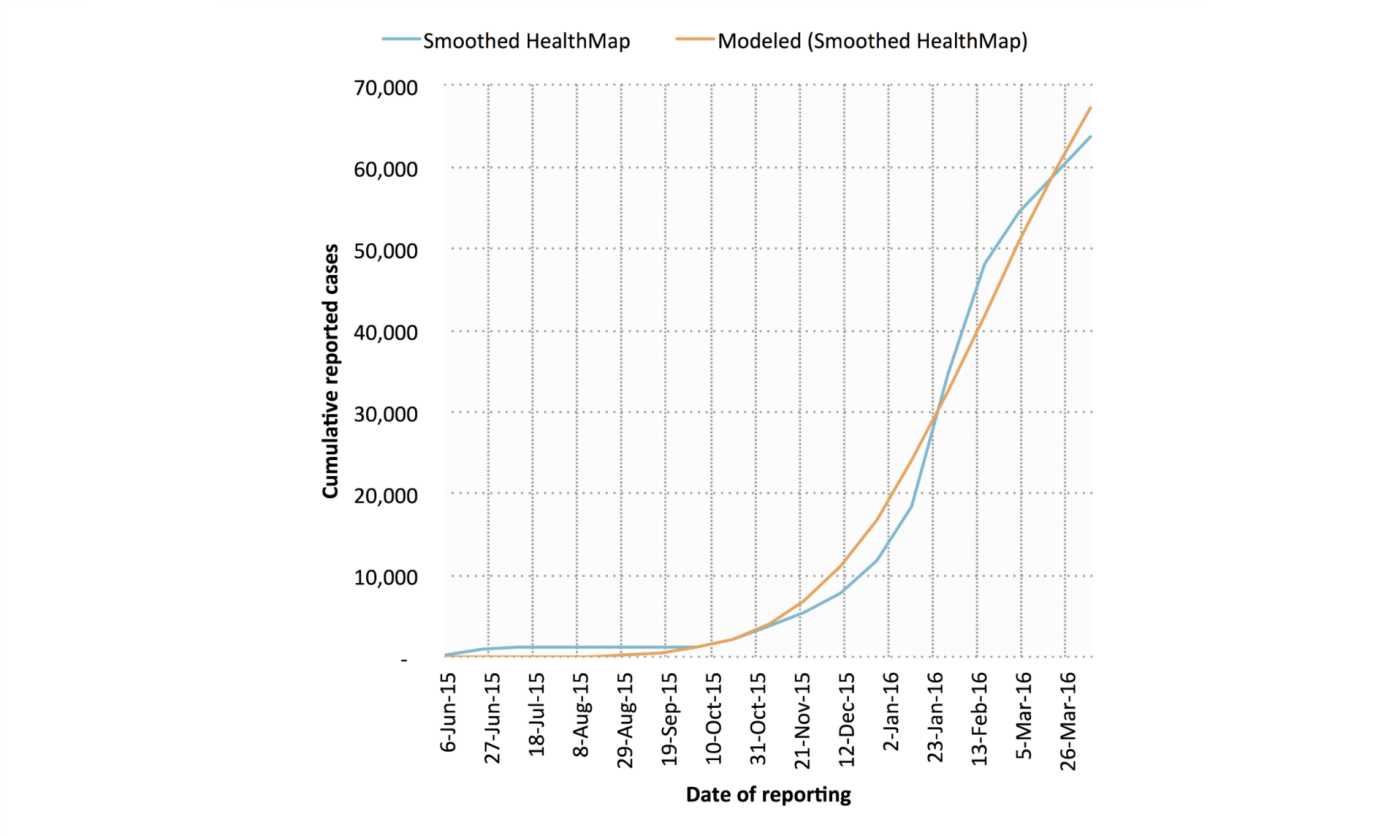
IDEA model fits against smoothed HealthMap cumulative case data for Zika virus disease in Colombia. A serial interval length of 17 days was used.

**Figure 5:**
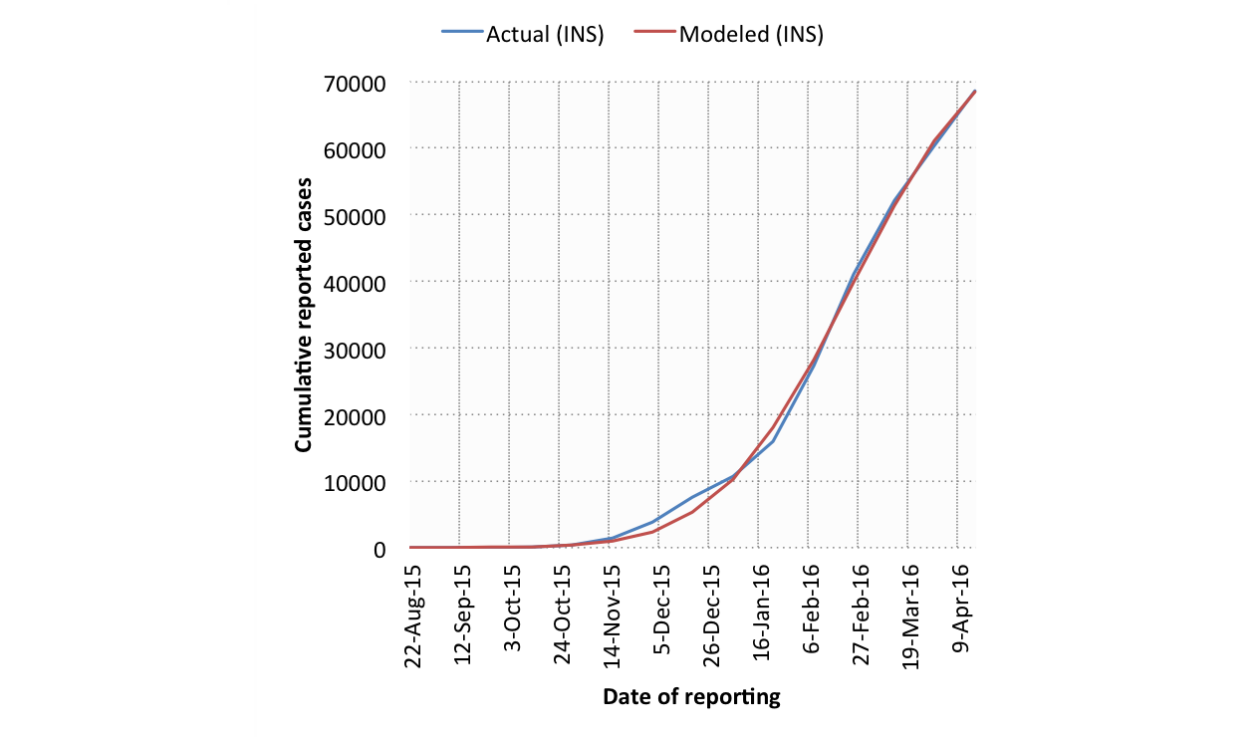
IDEA model fits against Instituto Nacional de Salud (INS) cumulative case data for Zika virus disease in Colombia. A serial interval length of 17 days was used.

Example model fits for both digital (smoothed HealthMap; SSD=1.47×10^8^) and traditional (INS; SSD=1.55×10^7^) cumulative case data are shown in Figures 4 and 5 (SI=17 days). In general, the traditional data model fits (mean SSD=1.76×10^7^) were superior to those derived from digital data (mean SSD=1.64×10^8^).

**Figure 6:**
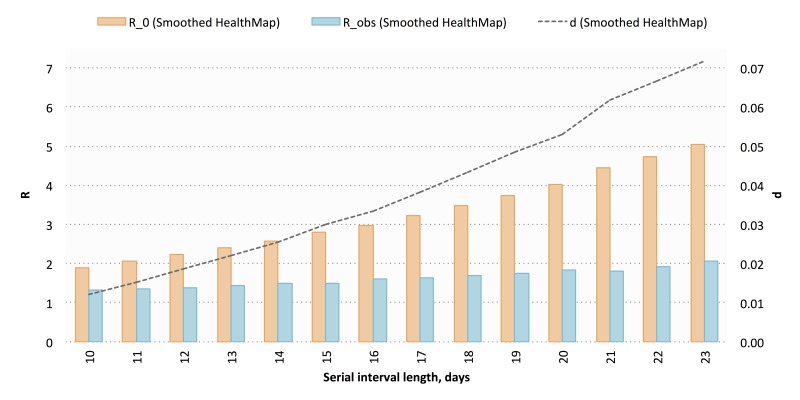
Modeled values for basic reproductive number (*R*_0_), discount factor (*d*), and observed reproductive number (*R*_obs_) using smoothed HealthMap cumulative case data. A total of 14 deterministic serial interval lengths were used; modeled values for each parameter are shown across all 14 serial interval lengths.

**Figure 7:**
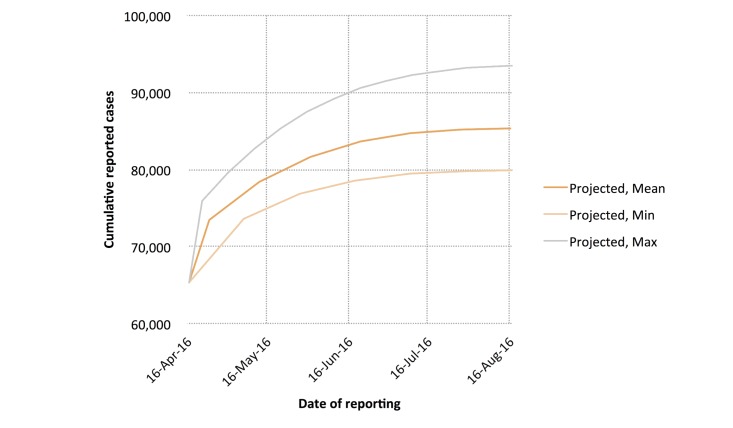
Cumulative case count projections using smoothed HealthMap cumulative case data. Projected minimum, maximum, and mean cumulative case counts are shown.

Using the digital (smoothed HealthMap) cumulative case counts, we estimated a mean *R*_0_ of 3.26 (range 1.91-5.05) and a mean *d* of 0.04 (range 0.01-0.07) across 14 deterministic serial interval lengths (range 10-23 days) (Figure 6). We then calculated a mean *R*_obs_ of 1.63 (range 1.31-2.05), a mean *I*_max_ of 85,546 cases (range 80,028-93,885 cases), and a mean *t*_max_ of 530 days (range 522-538 days; November 2016). Cumulative reported case projections using these modeled parameters are shown in Figure 7.

**Figure 8:**
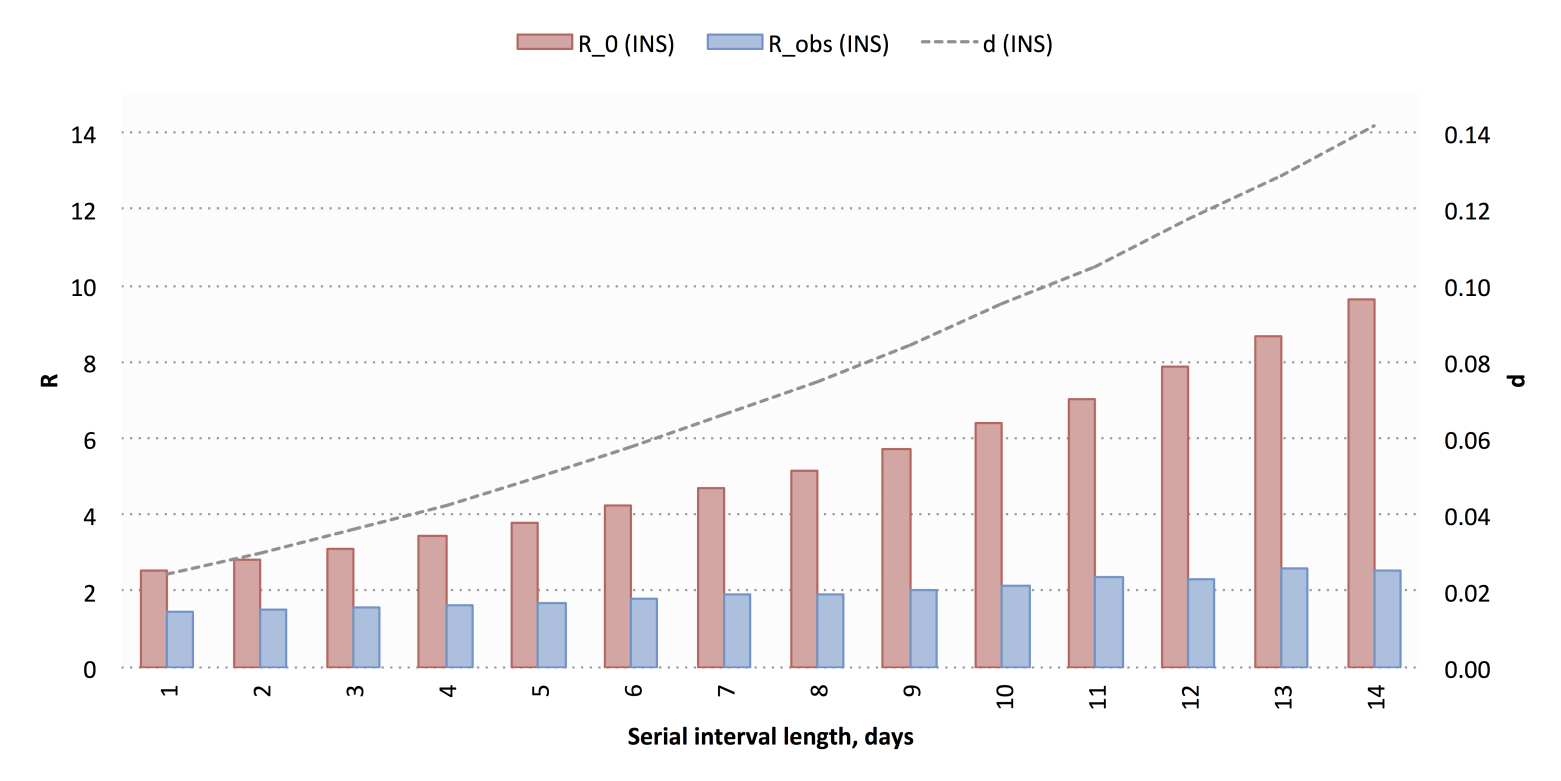
Modeled values for basic reproductive number (*R*_0_), discount factor (*d*), and observed reproductive number (*R*_obs_) using Instituto Nacional de Salud (INS) cumulative case data. A total of 14 deterministic serial interval lengths were used; modeled values for each parameter are shown across all 14 serial interval lengths.

**Figure 9:**
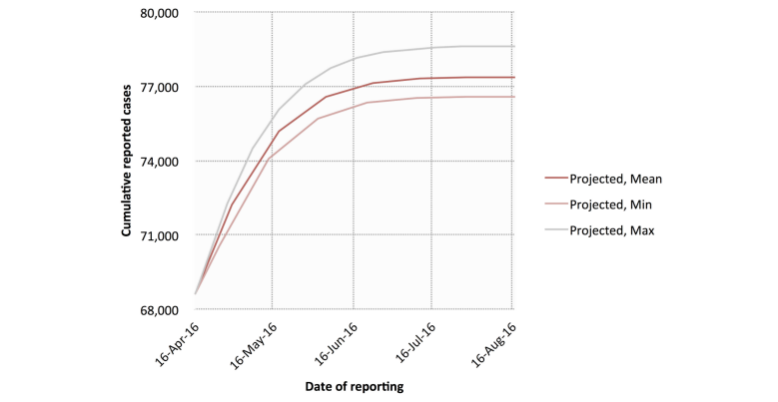
Cumulative case count projections using Instituto Nacional de Salud (INS) cumulative case data. Projected minimum, maximum, and mean cumulative case counts are shown.

The traditional (INS) data yielded a mean *R*_0_ of 5.36 (range 2.52-9.63) and a mean *d* of 0.07 (range 0.02-0.14) across 14 deterministic serial interval lengths (range 10-23 days) (Figure 8). Using these, we calculated a mean *R*_obs_ of 1.96 (range 1.45-2.58), a mean *I*_max_ of 77,386 cases (range 76,587-78,619 cases), and a mean *t*_max_ of 387 days (range 382-392 days; September 2016). Cumulative reported case projections using these modeled parameters are shown in Figure 9.

Although *R*_0_ values calculated using the traditional (INS) data were general higher than those calculated using digital (smoothed HealthMap) cumulative case counts (SSD=82.14), *R*_obs_ values were quite similar across data sources (SSD=1.84). As a result, the digital (smoothed HealthMap) and traditional (INS) cumulative case data produced similar mean %Vax values of 0.39 (range 0.24-0.51) and 0.49 (range 0.31-0.61), respectively.

## Discussion

When depletion of susceptible individuals due to infection (ie, via death or immunity-conferred recovery) is small relative to the total population, basic reproductive numbers obtained using the IDEA model are comparable to simple SIR-type models [19]. Although they are especially suitable for use in data-scarce settings, SIR-type models—and, by extension, the IDEA model—cannot easily incorporate global dynamics, such as the importation and exportation of infectious agents (ie, vectors and humans) or significant climate events (ie, El Niño and La Niña). Nevertheless, others have demonstrated that simple SIR-type models perform similarly to complex mechanistic models when describing the transmission dynamics of vector-borne and water-borne diseases in localized contexts [23,24]. As a result, the IDEA model is a reasonable method for analyzing nationwide transmission dynamics of Zika virus disease in Colombia.

As defined by the IDEA modeling method, *R*_0_ represents potential transmissibility of a given pathogen in a fully susceptible, naïve population; meanwhile, *R*_obs_ represents observed transmission in the face of existing interventions, as captured by *d* [2,18,19]. In this sense, the *R*_obs_ is similar to the effective reproductive number (*R*_t_), which represents transmissibility in a population that is not fully susceptible. Mean modeled estimates for *R*_0_ across both data sources were consistent with *R*_0_ estimates for Zika virus disease in French Polynesia and with *R*_0_ estimates for chikungunya and dengue [12,25,26]. Mean modeled estimates for *R*_obs_ were also comparable to *R*_t_ estimates for chikungunya and dengue [27,28]. To take into account the effects of ongoing transmission control efforts, *R*_obs_ was used instead of *R*_0_ to calculate %Vax.

In this study, we found that using the traditional (INS) data yielded higher *R*_0_ estimates than the digital (smoothed HealthMap) cumulative reported case counts. Nevertheless, because estimates for *d* were also higher, modeled ranges for *R*_obs_ and %Vax were comparable across both data sources. Similarly, the narrow range of possible case projections generated by the traditional (INS) data was largely encompassed by the wider range produced by the digital (smoothed HealthMap) cumulative reported case counts. Therefore, in the absence of traditional health care-based surveillance data, important epidemiologic parameters may be estimated using smoothed digital surveillance data as described here.

The methods used in this study are not without limitations. For both data sources, estimates for country-level case projections and *I*_max_ apply only to those that seek care; true caseloads are likely to be as much as five times higher than those that are reported [11,12]. Furthermore, because country-level data are utilized, in-country transmission heterogeneities are not captured. As geographic granularity of digital surveillance data improves, similar analyses should be conducted at smaller scales. Nevertheless, given that projection models are designed to serve as decision-support tools, estimating the number of cases that will report to hospitals and clinics over the next several months—even at the country level—is still valuable for the purposes of resource allocation. This may be especially pertinent with respect to diagnostic support for pregnant women presenting with clinical symptoms for Zika virus disease. To date, nearly 20% of all reported Zika virus disease cases in Colombia have been pregnant women; if the current rate holds, thousands more may be infected and seek care before the outbreak ends. However, the projections presented in this paper only apply in the event that circumstances remain unchanged (eg, no new interventions are put in place).

With improved compliance, vector suppression interventions (eg, elimination of standing water, exhaustive use of insect repellant) have the potential to bring this outbreak to a swift close, even in the absence of a vaccine. In the event that a viable vaccine can be developed before the outbreak ends, our estimates suggest that approximately half of the susceptible population would need to be immunized to confer herd immunity. Considering the growing body of evidence linking Zika virus infection during pregnancy to microcephaly in newborn babies, women of childbearing age should be given priority if the option becomes available [4,5].

Regardless of whether a vaccine reaches the market before the outbreak in Colombia ends, the data acquisition and modeling approach presented in this paper may still benefit other Zika-affected countries with limited capacity for government-implemented health care-based data collection. Although traditional surveillance data should be used preferentially, in its absence digital surveillance data can yield comparable estimates for key transmission parameters. It has been shown that digital surveillance data can be used retrospectively to assess transmission dynamics of well-understood pathogens (eg, *Vibrio cholerae*); however, our findings suggest that similar analyses can also be conducted in near real time for emerging infectious diseases [3]. Moreover, the epidemiologic parameters estimates from these analyses may be readily updated as new information emerges, enabling prospective tracking of transmission dynamics at the country level despite data scarcity.

Recent history has shown the need for rapid epidemiologic assessments to better inform intervention strategies in the face of a public health emergency. For effective evaluation of such interventions, baseline estimates for transmissibility—like those described in this study—must be established. Furthermore, changes in outbreak dynamics must be closely monitored in order to assess the impact of active interventions on disease transmission. Our approach offers an important alternative to guesswork based loosely on related diseases and previous outbreaks. Given the absence of traditional surveillance data and transmission heterogeneities across Central and South America, digital surveillance data can and should be used to conduct similar analyses for other Zika-affected countries in the months ahead.

## Abbreviations

%Vax: percentage of the susceptible population that would need to be immunized to eliminate transmission
d: discount factor
IDEA: Incidence Decay and Exponential Adjustment
I: cumulative incidence
Imax: final reported outbreak size
INS: Instituto Nacional de Salud
MERS: Middle East respiratory syndrome
R0: basic reproductive number
Robs: observed reproductive number
Rt: effective reproductive number
SARS: severe acute respiratory syndrome
SI: serial interval length
SSD: sum of squared differences
tmax: final reported outbreak duration
